# Unpacking the black box of training effectiveness: a structural equation modelling analysis

**DOI:** 10.1186/s41182-026-00993-9

**Published:** 2026-06-08

**Authors:** Ayako Masu, Tomoka Takano, Hirotsugu Aiga, Nobuaki Inoue, Sengdavy Xaypadith, Sadatoshi Matsuoka

**Affiliations:** 1 Bureau of Global Health Cooperation, Japan Institute for Health Security, 1-21-1 Toyama, Shinjuku-ku, Tokyo, 162-8655 Japan; 2 School of Tropical Medicine and Global Health, NEKKEN, Institute of Tropical Medicine, Nagasaki University, 1-12-4 Sakamoto, Nagasaki City, Nagasaki 852-8523 Japan; 3 Saitama Medical Center, Saitama Medical University, 1981 Kamoda, Kawagoe, Saitama 350-8550 Japan; 4 Ministry of Health in Lao People’s Democratic Republic, Ban Thatkhao, Sisattanack District, Vientiane, Laos; 5 Department of Human Resource Development, Bureau of Global Health Cooperation, Japan Institute for Health Security, 1-21-1 Toyama, Shinjuku-ku, Tokyo, 162-8655 Japan

**Keywords:** Capacity-building training, Japanese Official Development Assistance, Health workforce development, Structural equation modelling, Low- and middle-income countries

## Abstract

**Background:**

The health workforce shortage in low- and middle-income countries (LMICs) remains a critical challenge. The quality of health personnel impacts population health, particularly in LMICs. While development partners invest in training programmes to improve quality, evaluation of these programmes remains rare, with limited evidence on the structural and contextual factors influencing outcomes. This study aims to evaluate Japan’s Official Development Assistance (ODA) capacity-building training programmes conducted in Japan for LMIC health workforces and to identify key structural factors to guide future workforce development strategies.

**Methods:**

A cross-sectional questionnaire-based study was conducted from January to June 2023 in the Lao People’s Democratic Republic and Mongolia. A total of 148 health professionals who had participated in ODA training programmes between 2013 and 2020 were included. Exploratory factor analysis (EFA) was applied to 97 questionnaire items developed based on the training transfer literature, followed by Wilcoxon rank-sum tests and structural equation modelling (SEM) with robust estimation.

**Results:**

EFA identified six latent factors related to training effectiveness. Wilcoxon rank-sum tests showed that five factors—Feasible Action Plan, Collaborative Work Environment, Encouraging Learning Environment, Job-aligned Training and Readiness, and Altruistic Desire—were significantly associated with training effectiveness. During SEM refinement, two factors (Limited Authority and Altruistic Desire) were excluded due to non-significant effects or construct instability. The final model retained four factors, with Feasible Action Plan showing the strongest direct effect on training effectiveness (*β* = 0.72, *p* < 0.001). Collaborative Work Environment influenced training effectiveness indirectly through Job-aligned Training and Readiness and Feasible Action Plan. The model explained 45% of the variance in training effectiveness.

**Conclusions:**

Training effectiveness, defined as self-reported application of acquired knowledge, skills, and attitudes in the workplace, is shaped by interrelated factors operating before, during, and after training. The Feasible Action Plan emerged as the strongest direct predictor, highlighting the importance of translating learning into concrete and implementable actions. Collaborative and supportive workplace environments further facilitate this process. These findings underscore the importance of a holistic, process-oriented approach to ODA capacity-building training to enhance training transfer and sustain impact in LMIC settings.

**Supplementary Information:**

The online version contains supplementary material available at 10.1186/s41182-026-00993-9.

## Background

The global shortage of health workers, particularly in low- and middle-income countries (LMICs), remains a major barrier to achieving universal health coverage and Sustainable Development Goal 3 (SDG 3), which aims to ensure healthy lives and promote well-being for all. The availability and quality of health personnel are key determinants of population health outcomes [1]. To address this issue, multilateral and bilateral development partners have invested in training programmes to enhance knowledge, skills, and attitudes [2]. These initiatives are essential for improving healthcare quality and ultimately advancing health outcomes [1].

Despite growing recognition of the need to evaluate the effectiveness of training, many programmes lack rigorous evaluation. Scientifically verified studies on the effectiveness of training and its influencing factors remain rare, including in LMICs [3]. While some research has examined short-term outcomes, such as knowledge and skill acquisition, few studies have evaluated impacts on performance and population health [3–5].

Existing literature demonstrates that well-designed training programmes can significantly enhance health workers’ competencies and performance in LMICs [3, 6]. For example, a systematic review by Rowe et al. [3] found that training, when combined with strategies such as supervision and group problem-solving, improved healthcare provider practices, including patient assessment, diagnosis, and treatment. However, the effectiveness of training depends on multiple factors, including the relevance and quality of training content, appropriateness of delivery methods, and the broader organisational and health system context [7–9]. Limited evidence exists regarding the structural and contextual factors, such as sociocultural and organisational systems, that influence the effectiveness of training programmes, particularly those involving health workers from LMICs travelling to developed countries. These programmes require greater financial investment than in-country training, and their effectiveness may depend on the relevance of the content, the ability to transfer skills, and post-training support [2]. A comprehensive understanding of structural and contextual factors is crucial for optimising training programme design and ensuring efficient use of limited health workforce development resources [7–9].

To address these knowledge gaps, this study aimed to measure the overall effectiveness of development partner-supported training programmes for health workers from LMICs conducted in Japan and to identify key structural and contextual factors influencing their effectiveness. Using a structural equation modelling (SEM) approach, this study seeks to provide a comprehensive understanding of the complex relationships among the factors that contribute to the overall effectiveness of these training initiatives, while accounting for the specific context of training programmes involving international travel and higher costs. The findings of the study will serve as evidence for effective health workforce development strategies in LMICs and inform future training programme design to enhance their impact on population health outcomes.

## Methods

### Study design and setting

This cross-sectional questionnaire-based study was conducted in the Lao People’s Democratic Republic (Lao PDR) and Mongolia from January to June 2023. These countries were selected based on the authors’ established collaborative relationships with their respective Ministries of Health through Japan’s Official Development Assistance (ODA) technical cooperation projects. This context provides a valuable opportunity to examine factors influencing the effectiveness of international capacity-building initiatives, informing future policy and programme design. While cross-sectional designs inherently limit causal inference, this study aimed to explore complex associations among variables within a SEM framework.

### Sampling frame and participant selection

The target population was all health professionals who participated in Japan’s ODA capacity-building training programmes between 2013 and 2020. This period was selected as in-person training was suspended after 2020 due to COVID-19, and to minimise recall bias beyond 10 years. A purposive sampling strategy was employed to identify potential participants. Due to privacy and data protection policies, a comprehensive list of all past training participants from a Japanese development partner was not available to the research team. Instead, the sampling frame was constructed through a multi-pronged approach. We first utilised a list of past training participants held by our organisation, the National Centre for Global Health and Medicine (NCGM), which has been involved in implementing these ODA training programmes. Additionally, key personnel within the Ministries of Health in Lao PDR and Mongolia, with whom the authors had established long-term collaborative relationships through technical cooperation projects, assisted in identifying and contacting potential participants. Recognising the dispersed nature of the target population and the limitations in directly accessing a complete list, we further employed a snowball sampling technique. Initial participants identified through NCGM records and Ministry of Health referrals were asked to recommend other eligible colleagues who had also attended the ODA training programmes.

All identified individuals were invited to participate in the study.

Inclusion criteria were: (1) being a health professional (e.g., medical doctor, nurse, midwife, laboratory technician, public health officer) at the time of participation in the ODA training programme and remaining engaged in the health sector at the time of the survey; (2) having completed one of the designated ODA training programmes in Japan in the specified period (2013–2020); and (3) providing informed consent.

Exclusion criteria were: (1) having retired or no longer working in the health sector at the time of the survey; and (2) having incomplete training participation. Retired individuals were excluded because this study focused on training effectiveness as reflected in the application of acquired knowledge and skills within active workplace settings. Excluding retirees helped minimise recall bias and ensured that responses captured recent and ongoing opportunities for training transfer in organisational contexts.

Potential participants were contacted through various channels, and a total of 148 complete responses were obtained (Lao PDR: *n* = 58; Mongolia: *n* = 90). The reported 100% response rate (148 responses) refers to all individuals who were successfully contacted and agreed to participate in the survey, rather than the entire population of past trainees.

### Description of training programmes

The ODA capacity-building training programmes examined in this study covered a broad range of thematic areas related to health system strengthening in LMICs. Common training topics included health workforce development, nursing and midwifery practice, health service management, infection prevention and control, maternal and child health, quality improvement, and leadership and management in healthcare settings. These programmes typically combined lectures, group discussions, field visits, and practical exercises, with an emphasis on applying acquired knowledge and skills in participants’ home-country contexts. While the specific titles and curricula varied across programmes, they shared a common objective: strengthening participants’ capacity to improve health service delivery and health system performance upon returning to their workplaces. Participants in this study attended a variety of ODA capacity-building training programmes in Japan. For contextual understanding of the broad nature of these programmes, they can generally be categorised into two main types: theme-specific training and country-specific training. Table 1 provides a general overview of these categories.

**Table 1:** Overview of training programmes

	Theme-specific training	Country-specific training
Objective	To enhance knowledge and skills on specific health topics (e.g., infectious disease control, maternal and child health)	To achieve the goals of ongoing technical cooperation projects in the participants’ country
Target audience	Health professionals from multiple countries	Health professionals from a single country involved in a specific project
Language	English	Lao/Mongolian
Typical duration	2–4 weeks (average: 3 weeks)	1–3 weeks (average: 2 weeks)
Modality	On-site	On-site

This study’s primary objective was to examine the overall effectiveness of ODA capacity-building training programmes and the factors influencing it across all participants, rather than to conduct a comparative analysis of different training types. Therefore, specific data on which type of training (theme-specific or country-specific) each individual participant attended were not collected, as such a comparison was not within the scope of this research.

### Measurement instruments

#### Instrument adaptation

The questionnaire was adapted from Kozono and Ouchi’s validated training effectiveness scale [10], which was originally developed to assess training effectiveness from a training transfer perspective, with a particular focus on the application of acquired knowledge and skills in workplace settings. This instrument was selected over other available measures because it explicitly incorporates pre-training, during-training, and post-training factors that influence training transfer, aligning closely with the objectives of this study and the context of ODA capacity-building training programmes.

To ensure relevance to LMIC health sector settings, the questionnaire was reviewed and adapted by five experts in global health. It was translated into English by the authors, then into Lao and Mongolian by bilingual local assistants, and back-translated to ensure linguistic and conceptual equivalence.

#### Questionnaire structure

The questionnaire measured one dependent variable and a set of potential independent variables, which were initially conceptualised based on a comprehensive literature review on training transfer [3, 10–12]. All items were rated on a 6-point Likert scale (1 = strongly disagree to 6 = strongly agree). In the context of the Japanese ODA capacity-building training programmes examined in this study, participants were typically required to develop an action plan during the training. This action plan referred to a structured output that generally consisted of clearly defined activities, measurable indicators, achievable goals, proposed timelines, and consideration of required resources, tailored to participants’ priority public health or health system issues and intended for implementation after returning to their workplace.

The latent structure of these independent variables was subsequently explored through factor analysis, as detailed in the “Data analysis” section.

#### Dependent variable

The dependent variable, training effectiveness, was assessed using a composite score derived from five items adapted from previously published studies [10, 13], assessing the extent to which participants applied acquired knowledge, skills, and attitudes in the workplace. All five items are shown in the Supplementary Material (Questionnaire), from B-I-1 to B-I-5.

#### Independent variables—factors influencing training effectiveness

Ninety-seven items covering ten domains identified from the literature [3, 10–12], were grouped into four sections:

- Pre-training (13 items): training readiness (sample: “Before the training, I had already understood the objectives of the training”), organisational preparation or support (sample: “Before the training, I received an explanation about the contents of the training in detail by my organisation or boss”).
- During training (43 items): perceived content relevance (sample: “During the training, I felt that the training content was practical”), learning environment (sample: “The training had an atmosphere in which participants could talk freely and any mistake was allowable”).
- After training (6 items): opportunities to apply acquired knowledge (sample: “After the training, I was able to spend enough time applying what I had learned during the training”).
- Workplace environment (35 items): organisational and peer support for transfer of training (sample: “After the training, my boss and/or colleagues supported me in applying what I had learned during the training”).

### Data collection

Data were collected from January to May 2023 in Mongolia (90 samples) and from March to June 2023 in Lao PDR (58 samples) using a self-administered questionnaire incorporated into Google Forms. Before data collection, Japanese researchers trained a local assistant to facilitate the data collection. The assistant subsequently distributed the Google Forms to participants via email, WhatsApp, or conducted site visits to facilitate participation in the survey. The former was mainly applied to participants residing in remote areas, while the latter was adopted for those in urban areas where the local assistants lived.

### Data analysis

All statistical analyses were performed using STATA (BE18; STATACorp, College Station, TX, USA).

Descriptive statistics (means, standard deviations, frequencies) were calculated for all demographic variables and questionnaire items. The internal consistency reliability of the dependent variable scale was assessed using Cronbach’s alpha. Before SEM, the assumption of normality was assessed. Given the observed ceiling effect in 67.0% of the data (Lao PDR: 39.2%, Mongolia: 70.0%) and the ordinal nature of Likert-scale items, robust estimation methods suitable for non-normal distributions were employed in the SEM.

To identify the underlying latent structure of the 97 independent variable items within the context of Lao PDR and Mongolia, an exploratory factor analysis (EFA) was conducted. The analytical process began with a principal component analysis (PCA) to determine the number of factors to extract [14], with factors having eigenvalues greater than 1.0 initially considered for retention, in line with the Kaiser criterion [15]. The scree plot and cumulative contribution ratio were also used to determine the optimal number of factors.

After determining the number of factors, the main EFA was performed using principal axis factoring. A promax rotation was applied to allow for correlation between factors. For an item to be retained on a factor, a minimum loading of 0.4 was required [15].

Finally, the internal consistency of each identified factor was assessed using Cronbach’s alpha coefficients [16].

### Structural equation modelling

A two-step approach was adopted for SEM to examine the hypothesised relationships between the latent independent factors identified from EFA and training effectiveness. All models were estimated using maximum likelihood with robust standard errors to account for non-normal data distribution.

#### Measurement model assessment

While an explicit confirmatory factor analysis was not performed on a pre-defined theoretical structure in the traditional sense, the latent factors identified through EFA were treated as the measurement model for the subsequent structural analysis. The factor structure, item loadings, and reliability of these EFA-derived factors were evaluated within the SEM framework. Items with low factor loadings (e.g., below 0.6) or those causing instability in the model were considered for removal, with theoretical justification.

#### Structural model assessment

**Fig. 1:**
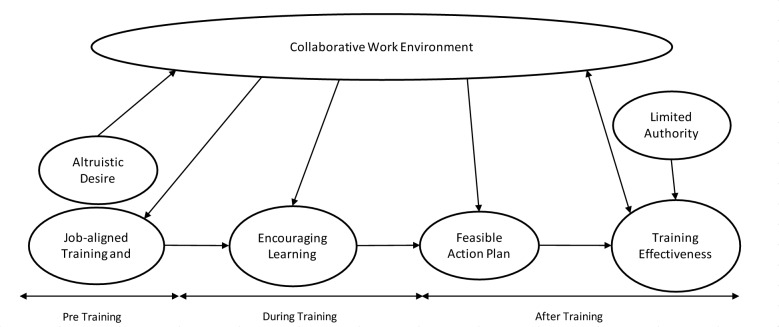
Initial hypothesised structural model based on factors identified through exploratory factor analysis (EFA). This figure illustrates the hypothesised relationships among latent factors influencing training effectiveness, organised according to a temporal perspective: pre-training (Job-aligned Training and Readiness, Altruistic Desire), during training (Encouraging Learning Environment), post-training (Feasible Action Plan, Limited Authority), and factors operating throughout the process (Collaborative Work Environment). Arrows represent hypothesised directional relationships between latent factors, indicating the assumed direction of influence rather than confirmed causality. The numbers adjacent to the arrows indicate standardised path coefficients [15]. For example, an arrow from Collaborative Work Environment to Job-aligned Training and Readiness represents the assumption that a supportive workplace environment before training enhances trainees’ perceived relevance of and readiness for training. Together, the model illustrates how conditions before, during, and after training are expected to interact to shape overall training effectiveness

Based on the identified and refined measurement model, a structural model was specified to test the hypothesised pathways from the independent factors to training effectiveness. The initial hypothesis guiding the structural model was formulated based on the temporal alignment of the EFA-derived factors (Pre-training, During-training, Post-training, Throughout) and their conceptual relationships, as presented in Fig. 1. The model was systematically refined by examining modification indices (MI) and theoretical considerations to improve model fit. Correlations between observed and/or latent variables were added only when supported by theoretical reasoning and substantial MI values (>5) [17, 18].

#### Model fit evaluation

The overall fit of the structural model was assessed using several goodness-of-fit indices: the root mean square error of approximation (RMSEA), the comparative fit index (CFI), and the standardised root mean square residual (SRMR). Acceptable fit was defined by RMSEA values ≤0.05, CFI values ≥0.95, and SRMR values ≤0.08 [18].

#### Non-parametric test

To further complement the findings from SEM and provide additional insight into the practical significance of the identified factors, a non-parametric Wilcoxon rank-sum test was employed. This test investigated whether the composite score for training effectiveness differed significantly between groups that scored high and low on each EFA-derived factor. Each factor was dichotomised at its median value to create these high- and low-score groups, addressing the non-normal distribution of the data. Because factor scores derived from EFA often include tied values at the median, stemming from identical response patterns on the original Likert scales, median-based dichotomisation may not yield equally sized high-score and low-score groups. In this study, individuals at the median were assigned to the higher-score group. Such an imbalance is an expected outcome of median split procedures when applied to distributions with ties or skewness.

## Results

### Participant characteristics and descriptive statistics

A total of 148 health professionals participated in this study, with 58 from Lao PDR and 90 from Mongolia. The demographic characteristics of the participants are summarised in Table 2. The majority of participants were male (73.6%), particularly in Mongolia, where the number of male respondents was twice that of female respondents. Participants represented various professional backgrounds, predominantly clinical officers (technical position) (29.7%) and administrative officers (management position) (29.1%).

**Table 2:** Participant characteristics

Characteristic	Lao PDR	Mongolia	Total
Total Participants, n (%)	58 (39.2%)	90 (60.8%)	148 (100%)
Female, n (%)	23 (15.5%)	16 (10.8%)	39 (26.4%)
Male, n (%)	35 (23.6%)	74 (50%)	109 (73.6%)
Administrative officer (Management position)*, n (%)	25 (17%)	18 (12%)	43 (29.1%)
Administrative officer (Technical position)*, n (%)	15 (10%)	14 (9.6%)	29 (19.6%)
Clinical officer (Management position)*, n (%)	14 (9%)	18 (12%)	32 (21.6%)
Clinical officer (Technical position)*, n (%)	4 (3%)	40 (27%)	44 (29.7%)
Dependent variable			
Training Effectiveness (Mean score, 1-6 scale), median (IQR)	6 (5-6)	6 (5-6)	6 (5-6)
Cronbach's alpha (Training Effectiveness)	0.99	0.99	0.99

*: Administrative officers are health sector officials primarily engaged in public health management, planning, or administrative functions, whereas clinical officers are health professionals primarily involved in direct clinical service delivery. Management position refers to roles that include supervisory, leadership, or decision-making responsibilities (e.g., department heads, unit managers, programme coordinators). Technical position refers to roles primarily focused on clinical, technical, or operational tasks without formal managerial authority

The dependent variable, training effectiveness, was assessed using a composite score derived from five items. The median composite score for training effectiveness was 6, with an interquartile range (IQR) of 5–6. The internal consistency reliability of this scale was high, with a Cronbach’s alpha coefficient of 0.99. Further preliminary analysis, such as PCA, confirmed that these five items loaded onto a single factor, explaining 65.9% of the total variance, thus supporting their aggregation into a single measure.

### Exploratory factor analysis of independent variables

An EFA was performed on 97 independent variable items to identify the underlying latent structure. Following the determination of the number of factors using PCA, guided by the eigenvalue criterion (≥1), visual inspection of the scree plot, and the cumulative contribution ratio, six distinct factors were retained.

The main EFA was then conducted using principal axis factoring with promax rotation. The six-factor structure cumulatively explained 89.9% of the total variance. The factor loadings for all retained items were ≥0.4.

The internal consistency reliability for each of these six factors was assessed using Cronbach’s alpha, which ranged from 0.78 to 0.96, indicating good to excellent reliability, except for Factor 6 (Cronbach’s alpha = 0.54) (Supplementary Table 1).

The six factors were conceptually interpreted and named as follows:

Factor 1: Feasible Action Plan (24 items). This reflects the extent to which participants developed concrete, realistic, and implementable plans during the training. This factor captures whether the action plans included clearly defined activities, measurable indicators, achievable goals, appropriate timelines, and consideration of available resources, and whether participants perceived these plans as directly applicable to their actual workplace context after returning from training.

Factor 2: Collaborative Work Environment (27 items). The factor refers to the workplace environment in participants’ country of origin (Lao PDR or Mongolia), including interactions with supervisors and colleagues at their place of employment, rather than relationships among co-participants or facilitators during the training programme in Japan.

Factor 3: Encouraging Learning Environment (20 items). The factor describes a positive learning environment that encourages participation and tolerates mistakes.

Factor 4: Job-aligned Training and Readiness (11 items). This relates to the match between training content and job needs, and the necessity of training.

Factor 5: Limited Authority (3 items). The factor indicates a lack of cooperation from superiors and insufficient authority to apply learnings.

Factor 6: Altruistic Desire (2 items). This represents a desire to prioritise and support others. In the initial EFA, this factor was originally specified with four items; however, the internal consistency was low (Cronbach’s alpha = 0.54) (Supplementary Table 1). Given the exploratory nature of the analysis, an iterative item-reduction process was undertaken to improve internal consistency and conceptual coherence, prioritising items with higher factor loadings and clearer theoretical alignment. This process resulted in a two-item factor with improved internal consistency (Cronbach’s alpha = 0.73), with retained item loadings of − 0.442 and − 0.462. Despite this improvement, the two-item factor was considered structurally fragile, as two-item latent constructs generally require strong theoretical justification or very high inter-item correlations to ensure stability. Accordingly, Factor 6 was treated as exploratory and subsequently excluded from the final structural equation model due to concerns about construct stability and its limited contribution to explained variance.

Item refinement was guided not only by internal consistency metrics such as Cronbach’s alpha, but also by conceptual considerations, including overlap with other factors and logical coherence within each construct. Given the exploratory nature of the analysis and the complex, multi-dimensional characteristics of organisational and contextual factors, a relatively large number of items were retained for several factors to ensure adequate conceptual coverage. While high Cronbach’s alpha values may partly reflect item redundancy or response patterns, further item reduction was intentionally limited at this stage to avoid premature loss of potentially relevant dimensions. The full questionnaire used for data collection is provided in the Supplementary Materials to allow reference to item wording and structure.

From a temporal perspective, these factors broadly aligned with different stages of the training process (Table 3): pre-training (Job-aligned Training and Readiness, Altruistic Desire), during training (Encouraging Learning Environment), post-training (Feasible Action Plan, Limited Authority), and throughout the entire period (Collaborative Work Environment). These identified factors subsequently formed the latent independent variables in the SEM.

**Table 3:** Summary of identified factors and their reliability

Factor category	Factor name	Number of items	Cronbach’s alpha
Pre-training	Factor 4: Job-aligned Training and Readiness	11	[0.92]
	Factor 6: Altruistic Desire	2	[0.73]
During training	Factor 3: Encouraging Learning Environment	20	[0.92]
Post-training	Factor 1: Feasible Action Plan	24	[0.96]
	Factor 5: Limited Authority	3	[0.78]
Throughout the entire period	Factor 2: Collaborative Work Environment	27	[0.96]

### Non-parametric test

To provide complementary insights into the practical relevance of the six identified factors, their relationship with the training effectiveness was examined. For each factor, participants were divided into a ‘high-score’ group and a ‘low-score’ group at its median score. A Wilcoxon rank-sum test was then performed to compare training effectiveness between these two groups.

The results indicated significant differences (*p* < 0.0005) between high- and low-score groups for five of the six factors (Table 4). Specifically, participants with higher scores on Feasible Action Plan, Collaborative Work Environment, Encouraging Learning Environment, Job-aligned Training and Readiness, and Altruistic Desire exhibited significantly greater training effectiveness. The only factor that did not show a significant difference was Limited Authority.

**Table 4:** Wilcoxon rank-sum test results: comparison of training effectiveness by factor score group

	Factor 1		Factor 2		Factor 3		Factor 4		Factor 5		Factor 6	
	Feasible Action Plan		Collaborative Work Environment		Encouraging Learning Environment		Job-aligned Training and Readiness		Limited Authority		Altruistic Desire	
Sample	H	L	H	L	H	L	H	L	H	L	H	L
	(*n* = 77)	(*n* = 71)	(*n* = 78)	(*n* = 70)	(*n* = 75)	(*n* = 73)	(*n* = 77)	(*n* = 71)	(*n* = 83)	(*n* = 65)	(*n* = 103)	(*n* = 45)
Rank sum	7407.5	3618.5	7081.5	3944.5	6967.5	4058.5	7307.5	3718.5	6185.5	4840.5	8486.5	2539.5
Expected	5736.5	5289.5	5811.0	5215.0	5587.5	5438.5	5736.5	5289.5	6183.5	4842.5	7673.5	3352.5
*z* value	6.568		4.997		5.42		6.175		0.008		3.47	
*p* value	<0.0001		<0.0001		<0.0001		<0.0001		<0.9937		<0.0005	

Participants were divided into high-score (H) and low-score (L) groups based on the median value of each factor score

### Structural equation modelling

The SEM was constructed based on the six latent factors identified through EFA and the single composite measure of training effectiveness. The initial specified model, reflecting the temporal and conceptual relationships (Fig. 1), showed suboptimal goodness-of-fit.

A systematic model refinement process was undertaken. First, Factor 5, Limited Authority, was removed from the model due to its non-significant effect in preliminary analyses (i.e. the Wilcoxon rank-sum test) in the refined structural model. Second, Factor 6, Altruistic Desire, was also excluded due to its instability as a latent construct (comprising only two items) [15] and its limited contribution to explaining variance in the overall model.

Following these exclusions, the remaining four factors (Feasible Action Plan, Collaborative Work Environment, Encouraging Learning Environment, Job-aligned Training and Readiness) were utilised in the refined structural model. Further optimisation involved raising the loading cutoff for observed variables to 0.6 and applying MI to establish theoretically justifiable covariances between highly correlated variables (MI range: 5–57.7). For instance, covariances were added between specific observed variables/latent variables with theoretical justification, e.g., ‘My workplace usually has an organizational culture in which coworkers actively acquire new knowledge, skills and know-how that seem to be useful’ and ‘During the training, my boss understood the objectives of the training’ within Collaborative Work Environment as they capture similar aspects of peer support.

**Fig. 2:**
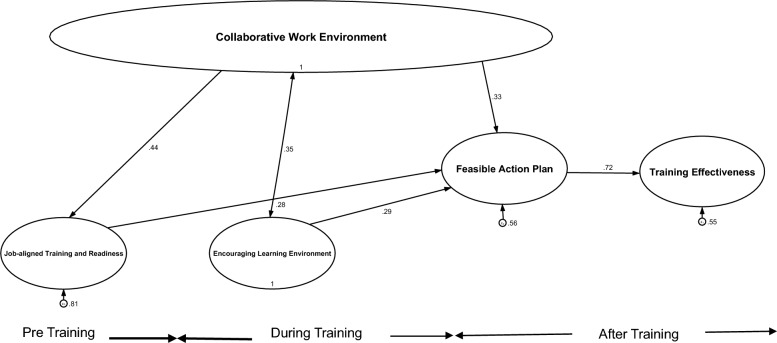
Final refined structural equation model with standardised path coefficients

The refined SEM model achieved acceptable goodness-of-fit indices (Fig. 2): RMSEA = 0.042 (90% confidence interval (CI): 0.031–0.051), CFI = 0.965, and SRMR = 0.072, indicating a good representation of the relationships between the remaining factors and training effectiveness [18]. The same model with covariances is presented in Supplementary Figure 1.

Figure 2 illustrates the standardised path coefficients and their significance for the refined structural model. All hypothesised paths were statistically significant (*p* < 0.001).

Collaborative Work Environment was positively associated with Job-aligned Training and Readiness (*β* = 0.44, *p* < 0.001). It was also associated with Feasible Action Plan (*β* = 0.33, *p* < 0.001). In addition, Encouraging Learning Environment was positively associated with Feasible Action Plan (*β* = 0.29, *p* < 0.001). Feasible Action Plan had the strongest direct positive effect on Training Effectiveness (*β* = 0.72, *p* < 0.001). Finally, Collaborative Work Environment and Encouraging Learning Environment also exhibited a significant positive correlation (*r* = 0.35, *p* < 0.001). The model explained 45% of the variance in Training Effectiveness.

## Discussion

This study aimed to comprehensively analyse the factors influencing the effectiveness of ODA capacity-building training programmes for health professionals in Lao PDR and Mongolia, employing a multifaceted approach including EFA, non-parametric tests, and SEM. The findings offer several key insights into optimising training design and implementation in similar contexts. First, our EFA initially identified six latent factors, which conceptually aligned with distinct temporal stages of the training process: pre-training (Job-aligned Training and Readiness, Altruistic Desire), during-training (Encouraging Learning Environment), post-training (Feasible Action Plan, Limited Authority), and throughout the entire period (Collaborative Work Environment). This temporal alignment, as hypothesised in Fig. 1, underscored the necessity of adopting a comprehensive, long-term perspective when evaluating and designing training programmes, rather than focusing solely on isolated stages. This finding supports the view that training effectiveness is not determined solely by the training event itself. Rather, it reflects a combined influence of factors operating before, during, and after the intervention, as suggested by established training transfer and evaluation frameworks [3, 10, 13, 19]. Our complementary non-parametric Wilcoxon rank-sum test provided initial evidence that these factors have real-world importance, demonstrating that their effects are not only statistically significant but also practically meaningful. Notably, Limited Authority was the only factor that did not show a statistically significant difference in training effectiveness between the high- and low-scoring groups. This may be attributed to the fact that the majority of participants were managers and held some authority. This observation justified its subsequent exclusion in the final structural model. Additionally, Altruistic Desire was also excluded from the final model due to its construct instability and limited explanatory power, indicating that while potentially relevant, its direct impact on the pathways to training effectiveness was not significant within this refined framework.

The SEM confirmed and elucidated the complex interrelationships among the identified factors and their impact on training effectiveness, providing a nuanced understanding that extends beyond simple correlations. The final refined model (Fig. 2) achieved a good fit, highlighting several critical pathways. Crucially, Collaborative Work Environment, defined as the workplace context in participants’ home country, emerged as a foundational factor influencing Job-aligned Training and Readiness (*β* = 0.44, *p* < 0.001). This suggests that a supportive peer and supervisory environment within the workplace may play a vital role in ensuring trainees perceive the training as relevant to their jobs and are adequately prepared to learn before training, consistent with prior evidence on organisational support and training readiness [3, 7, 10]. This finding highlights the importance of fostering a collaborative culture as a prerequisite for effective training. It is crucial not only for trainees themselves, but also for their colleagues and superiors, to understand in advance the relevance and necessity of the training programme to their work.

Furthermore, the Feasible Action Plan was found to be a central mediator, influenced by three key factors: Collaborative Work Environment (*β* = 0.33, *p* < 0.001), Encouraging Learning Environment (*β* = 0.29, *p* < 0.001), and Job-aligned Training and Readiness (*β* = 0.28, *p* < 0.001). These findings indicate that when trainees are well-prepared and supported by both their home-country workplace and the training environment in Japan, they are better positioned to develop practical action plans for applying the skills they have learned. Notably, Collaborative Work Environment and Encouraging Learning Environment also exhibited a significant positive correlation (*r* = 0.35, *p* < 0.001), indicating that these two environmental factors are closely intertwined and tend to coexist or mutually reinforce one another, as suggested by prior studies highlighting the interaction between learning environments and workplace support for training transfer [7, 9, 20].

Most significantly, the path from Feasible Action Plan to Training Effectiveness exhibited the strongest relationship in the model (*β* = 0.72, *p* < 0.001). This underscores that the development of a concrete, achievable plan is the most critical proximal predictor of actual training outcomes. For participants from Mongolia and Lao PDR, this implies that the ability to bridge their learning in Japan with the collaborative realities of their home organisations through a structured action plan is the ultimate key to sustained training impact. Simply attending training is insufficient; the ability to translate learning into concrete, actionable steps is key to achieving desired outcomes. Rather than being treated as a routine training output, the action plan should be recognised as a practical mechanism for translating learning into workplace implementation. The model successfully explained 45% of the factors influencing Training Effectiveness. While this demonstrates a moderate degree of explanation, it also suggests that other unmeasured factors contribute significantly to the remaining 55% of the factor. Furthermore, Job-aligned Training and Readiness had an *r*^2^ of 0.19, indicating that 81% of its variance is not explained by Collaborative Work Environment, underscoring the need to explore additional factors related to trainee readiness in future research.

These findings extend previous research on training effectiveness [20–22] by providing a comprehensive structural model that integrates key pre-, during-, and post-training factors within a specific LMIC context, refined through an EFA-driven SEM approach to analyse such structural factors for ODA capacity-building training in the health sector. To our knowledge, this is the first study to employ this approach in this specific context.

### Policy implications

The results of this study have significant implications for development partners (e.g., Japan International Cooperation Agency, World Health Organization, other aid agencies) and national government agencies involved in ODA capacity-building for health professionals.

In the pre-training phase, it is important to recognise that workplace support must begin before the trainee departs for the programme to establish Job-aligned Training and Readiness. This involves promoting supervisor support and peer collaboration even before training begins, thereby creating an environment in which trainees are more likely to perceive the training as relevant and better prepared to learn. Complementary to this, thorough needs assessments and a transparent trainee selection process should be prioritised. This initial investment creates a fertile ground for subsequent learning and transfer.

During the training phase, an encouraging learning environment should be fostered in which participants feel safe to ask questions, make mistakes, and actively engage. Training facilitators should be equipped with skills to create such an atmosphere. The development of feasible action plans with clear objectives and indicators should be emphasised to guide participants in translating theoretical knowledge into practical application.

In the post-training phase, emphasis should be placed on supporting the implementation, monitoring, and adaptation of the feasible action plans developed during training. Policy-makers and programme managers should establish mechanisms for post-training follow-up, such as progress reviews, practical troubleshooting, and opportunities to revise action plans in response to local workplace realities. Continued support after training is essential to sustain the application of newly acquired knowledge and skills in practice. In this regard, the non-significant impact of Limited Authority suggests that informal support and encouragement may matter more than formal hierarchical power alone in facilitating training transfer [3, 8, 21].

### Limitations of the study

This study, while offering novel insights, is subject to several limitations that warrant consideration for interpretation and future research.

First and foremost, while the ODA training programmes were broadly categorised into theme-specific and country-specific modalities, data on the specific type of training each participant attended were not collected. This methodological constraint precluded our ability to conduct comparative analyses on the effectiveness or influencing factors between these two distinct training types. Understanding these potential differences is critical for future research, particularly because such differences would necessitate distinct policy approaches and interventions for each context.

Second, limitations in the sample’s representativeness should be carefully considered, as the combined use of purposive and snowball sampling, alongside difficulties in accessing a complete sampling frame, may result in a sample that is not fully representative of all ODA training alumni. This non-random sampling approach may thus introduce selection bias. In addition, participant identification through Ministry of Health referrals, together with the authors’ institutional involvement in some of the training programmes, may have introduced socially desirable or overly positive responses. Although anonymous, self-administered questionnaires were used to reduce this risk, such bias cannot be fully excluded.

Third, sample size constraints necessitated combining data from Lao PDR and Mongolia to meet the generally recommended minimum for SEM analysis [23]. Consequently, our findings represent a pooled analysis, preventing the identification of country-specific trends or distinctions. Furthermore, the disparity in sample sizes between the two countries might have introduced a potential bias towards the larger Mongolian sample.

Fourth, although efforts were made to refresh participants’ memories by asking them to review their training before completing the questionnaire, a substantial amount of time had passed since training completion for some respondents. As a result, recall bias could not be fully eliminated, and earlier participants may have retrospectively reinterpreted training outcomes, potentially attenuating or amplifying perceived effectiveness.

Fifth, as this study employed a cross-sectional, retrospective design, it was not possible to examine temporal changes in perceptions of training effectiveness. Future longitudinal studies would be better suited to capture how perceived and actual training effectiveness evolve at different stages following training completion.

Sixth, a notable limitation concerns the explained variance in the structural model. While the final model accounted for 45% of the variance in Training Effectiveness, a substantial proportion (55%) remains unexplained by the included factors. More critically, Job-aligned Training and Readiness had only 19% of its variance explained by Collaborative Work Environment. These findings indicate that other unmeasured factors—such as individual motivation, specific features of training content, broader organisational culture beyond the immediate work environment, or external contextual variables—are likely to play a significant role in shaping training effectiveness. Future research should aim to identify and incorporate these additional factors to build a more comprehensive and predictive model. In addition, the relatively large number of items retained for some factors and the resulting high internal consistency may indicate item redundancy or shared response tendencies, which should be further examined through confirmatory analyses in future studies. Finally, ceiling effects were observed in a substantial number of questions, particularly prominent in the Mongolian dataset, where participant evaluations clustered at the highest end of the measurement scale. This phenomenon limits the capture of granular individual differences and response variability. While this tendency might partly reflect cultural characteristics favouring positive self-expression [24], it primarily highlights a measurement challenge. To address this, we employed non-parametric methods where appropriate. For future studies, we recommend a more refined approach to measurement scale development, incorporating comprehensive pretesting and context-specific question reformulation [24] to capture the full range of responses better and minimise ceiling effects across diverse cultural settings.

Despite these limitations, this study provides empirically grounded insights into key structural and contextual factors that influence the perceived effectiveness of ODA capacity-building training programmes, and offers practical guidance for improving training design, implementation, and post-training support in similar low- and middle-income country contexts.

## Conclusions

This study highlights that effective ODA training for health professionals requires a holistic, process-oriented approach, integrating strategic planning before training, effective facilitation during training, and robust support within the workplace, especially through collaborative environments, to ensure the successful implementation of feasible action plans. These insights can serve as a valuable guide for designing more impactful and sustainable capacity-building initiatives in LMICs.

## Supplementary Information

Additional file 1.

Additional file 2.

## Data Availability

The data are available from the corresponding author upon reasonable request.

## Abbreviations

CFI: Comparative fit index
EFA: Exploratory factor analysis
Lao PDR: Lao People’s Democratic Republic
LMICs: Low- and middle-income countries
MI: Modification indices
ODA: Official Development Assistance
PCA: Principal component analysis
RMSEA: Root mean square error of approximation
SEM: Structural equation modelling
SRMR: Standardised root mean square residual
