# Supplementary material for: Unpacking the black box of training effectiveness: a structural equation modelling analysis
Source: Trop Med Health. 2026 Jun 8;54:139. doi: 10.1186/s41182-026-00993-9 (PMC13470938; doi:10.1186/s41182-026-00993-9)
Supplement: Supplementary file 1 [file 41182_2026_993_MOESM1_ESM.docx]

**Supplementary Figure 1 Model with Modification Indices**

| **Feasible Action Plan** | |
| --- | --- |
| 1 | Before the training, I felt that the training would be helpful (/useful) for my subsequent work. |
| 2 | Before the training, I prepared myself for the training by reading materials and working on pre-assignments. |
| 3 | During the training, I was able to develop my/our action plan, which would be carried out with high feasibility. |
| 4 | During the training, I could set appropriate outcome indicators in my/our action plan. |
| 5 | When developing my/our action plan, it had a high degree of completion. |
| 6 | I was able to carry it out when developing my/our action plan. |
|  |  |
| **Collaborative Work Environment** | |
| 1 | After the training, the working environment (e.g., tools and equipment) was in place for me to apply the knowledge and skills I had learned. |
| 2 | My workplace usually has an organizational culture where coworkers help each other when they are in trouble. |
| 3 | My workplace usually has an organizational culture in which coworkers actively acquire new knowledge, skills, and know-how that are useful. |
| 4 | My workplace usually has a free atmosphere in which anyone can talk freely. |
| 5 | My workplace usually has an organizational culture in which coworkers show me a positive attitude toward applying what I learned in any training. |
| 6 | My coworkers and I usually have opportunities to do group work to solve various issues. |
| 7 | My workplace has a policy that a training participant should transfer what he/she learned during training to coworkers after the training. |
| 8 | The scope of work for people at my workplace is not excessively fixed, and it is relatively easy for us to transfer knowledge and skills to coworkers. |
| 9 | In my workplace, the job description of each worker is clear enough, and personnel evaluation is done based on the description. |
| 10 | During the training, my boss understood the objectives of the training. |
| 11 | Before the training, my boss and/or colleagues reinforced my motivation for attending the training. |
| 12 | After the training, my boss and/or colleagues supported me in applying what I had learned during the training. |
| 13 | My boss usually conducts supportive supervision for his/her subordinates, including me. |
| 14 | The relationship between my boss and me is good. |
| 15 | My boss usually facilitates or supports my capacity building. |
| 16 | A third party usually checks whether people at my workplace practice what they learned in any training. |
| 17 | Before the training, my boss expected that my awareness, knowledge, and skills would be improved by the training. |
|  |  |
| **Encourage Learning Environment** | |
| 1 | I could attend the training in a relaxed mood. |
| 2 | I could actively attend the training. |
| 3 | The training had an atmosphere where participants could talk freely, and any mistake was allowable. |
| 4 | During the training, lecturers gave feedback about my speech and behaviour. |
| 5 | The teaching skill level of lecturers (speaking speed, clear and well-designed explanations) was high enough. |
|  |  |
| **Job-aligned and Readiness** | |
| 1 | During the training, I made an effort to understand the training content. |
| 2 | Before the training, I had already perceived the need for the training that I would participate in. |
| 3 | Before the training, I felt that the training would be helpful for my subsequent work. |
| 4 | Before the training, the needs of the workplace corresponded to the training contents. |
| 5 | Before the training, I had already understood the objectives of the training. |
