# Supplementary material for: Unpacking the black box of training effectiveness: a structural equation modelling analysis
Source: Trop Med Health. 2026 Jun 8;54:139. doi: 10.1186/s41182-026-00993-9 (PMC13470938; doi:10.1186/s41182-026-00993-9)
Supplement: Supplementary file 2 [file 41182_2026_993_MOESM2_ESM.docx]

Supplementary Table 1. Detailed factor loadings, uniqueness, summary of identified factors and their reliability

| Item description | **Factor 1** | **Factor 2** | **Factor 3** | **Factor 4** | **Factor 5** | **Factor 6** | **Uniqueness** | **Factor name** | **Cronbach's alpha** |
| --- | --- | --- | --- | --- | --- | --- | --- | --- | --- |
| Before the training, I prepared myself for the training by reading materials and working on pre-assignments. | 0.847 | 0.180 | 0.023 | -0.072 | -0.051 | -0.153 | 0.216 | Feasible action plan | 0.96 |
| During the training, I was able to develop my/our action plan, which would be carried out with high feasibility. | 0.793 | 0.070 | -0.015 | 0.050 | 0.016 | -0.079 | 0.307 |  |  |
| When developing my/our action plan, it had a high degree of completion. | 0.763 | 0.169 | -0.077 | 0.125 | -0.041 | -0.090 | 0.278 |  |  |
| During the training, I could set appropriate outcome indicators in my/our action plan. | 0.709 | 0.146 | -0.065 | 0.110 | 0.048 | -0.050 | 0.349 |  |  |
| I was able to carry it out when developing my/our action plan. | 0.705 | 0.036 | 0.112 | 0.129 | -0.056 | -0.199 | 0.318 |  |  |
| When developing my/our action plan, I was motivated to carry it out. | 0.661 | -0.006 | 0.169 | 0.144 | -0.117 | -0.172 | 0.357 |  |  |
| Before the training, I felt that the training would be helpful (/useful) for my subsequent work. | 0.653 | -0.007 | 0.161 | 0.089 | 0.013 | 0.044 | 0.354 |  |  |
| After the training, I had an opportunity in my workplace to look back on the implementation status (progress, achievements) of what I had learned during the training. | 0.582 | 0.518 | -0.224 | 0.075 | -0.116 | 0.094 | 0.246 |  |  |
| After the training, I had an opportunity to apply in my workplace what I had learned during the training. | 0.577 | 0.594 | -0.254 | -0.082 | -0.144 | 0.090 | 0.281 |  |  |
| During the training, I could apply concepts, theories, and methods I learned to my workplace. | 0.569 | -0.052 | 0.157 | 0.075 | 0.038 | 0.240 | 0.409 |  |  |
| During the training, the training content was practical. | 0.555 | -0.033 | 0.250 | 0.060 | 0.009 | -0.024 | 0.465 |  |  |
| After the training, I tried to spend time applying in my workplace what I had learned. | 0.553 | 0.140 | -0.082 | 0.152 | 0.016 | 0.263 | 0.414 |  |  |
| During the training, a leader among the participants promoted my understanding. | 0.544 | 0.102 | 0.154 | -0.140 | 0.174 | 0.149 | 0.471 |  |  |
| At the end of the training, I could master the training content well. | 0.502 | 0.022 | 0.333 | 0.026 | -0.109 | 0.093 | 0.424 |  |  |
| During the training, I could organize and explain any issues in my workplace. | 0.496 | -0.067 | 0.187 | 0.097 | 0.101 | 0.257 | 0.433 |  |  |
| I could understand the concepts, theories, and methods included in the training content during the training. | 0.494 | -0.071 | 0.359 | 0.086 | -0.024 | 0.168 | 0.352 |  |  |
| During the training, the training content corresponded to my job description. | 0.470 | 0.036 | 0.196 | 0.058 | -0.013 | 0.230 | 0.480 |  |  |
| I exchanged information unrelated to the training content with other participants during the training. | 0.463 | -0.060 | 0.325 | -0.327 | 0.288 | 0.031 | 0.527 |  |  |
| During the training, I could learn some things from other participants. | 0.452 | -0.008 | 0.386 | -0.244 | 0.284 | 0.109 | 0.411 |  |  |
| After the training, I was able to spend enough time applying what I had learned during the training. | 0.443 | 0.462 | -0.109 | 0.068 | -0.003 | 0.120 | 0.396 |  |  |
| During the training, I had an opportunity to work with competent participants. | 0.419 | -0.056 | 0.218 | -0.030 | 0.314 | 0.276 | 0.440 |  |  |
| During the training, other participants positively influenced my motivation to learn. | 0.417 | -0.137 | 0.341 | -0.009 | 0.221 | 0.117 | 0.495 |  |  |
| I exchanged information on the training content with other participants during the training. | 0.412 | 0.031 | 0.577 | -0.331 | -0.028 | -0.038 | 0.446 |  |  |
| At the end of the training, I could understand the training content well. | 0.409 | -0.065 | 0.508 | 0.006 | -0.175 | 0.065 | 0.405 |  |  |
| My coworkers and I usually have opportunities to do group work to solve various issues. | 0.157 | 0.860 | -0.036 | -0.102 | -0.113 | -0.228 | 0.297 | Collaborative work environment | 0.96 |
| My workplace usually has an organizational culture in which coworkers actively acquire new knowledge, skills, and know-how that are useful. | 0.075 | 0.841 | -0.053 | -0.009 | 0.002 | -0.028 | 0.280 |  |  |
| In my workplace, the job description of each worker is clear enough, and personnel evaluation is done based on the description. | -0.093 | 0.824 | -0.034 | 0.007 | -0.044 | 0.105 | 0.363 |  |  |
| My workplace usually has an organizational culture in which coworkers show me a positive attitude toward applying what I learned in any training. | 0.072 | 0.822 | 0.176 | -0.150 | -0.045 | -0.001 | 0.267 |  |  |
| My workplace has a policy that a training participant should transfer what he/she learned during training to coworkers after the training. | -0.027 | 0.814 | 0.055 | -0.046 | -0.032 | 0.032 | 0.357 |  |  |
| My boss usually facilitates or supports my capacity building. | -0.207 | 0.808 | 0.071 | 0.123 | 0.065 | -0.127 | 0.314 |  |  |
| My workplace usually has a free atmosphere in which anyone can talk freely. | 0.145 | 0.768 | -0.052 | -0.088 | 0.057 | 0.004 | 0.353 |  |  |
| The relationship between my boss and me is good. | -0.137 | 0.746 | 0.214 | -0.016 | -0.003 | 0.038 | 0.386 |  |  |
| My workplace usually has an organizational culture where coworkers help each other when they are in trouble. | 0.106 | 0.739 | 0.027 | 0.010 | -0.003 | 0.086 | 0.324 |  |  |
| My boss usually conducts supportive supervision for his/her subordinates, including me. | -0.021 | 0.733 | 0.021 | 0.148 | 0.022 | -0.105 | 0.365 |  |  |
| My workplace has a rule or system that a training participant should report what he/she learned during training to coworkers after the training. | -0.118 | 0.706 | 0.207 | -0.042 | 0.005 | -0.032 | 0.472 |  |  |
| After the training, my boss and/or colleagues supported me in applying what I had learned during the training. | 0.247 | 0.702 | -0.054 | 0.007 | -0.109 | 0.129 | 0.318 |  |  |
| During the training, my boss understood the objectives of the training. | 0.052 | 0.685 | 0.035 | 0.087 | -0.152 | 0.014 | 0.463 |  |  |
| The scope of work for people at my workplace is not excessively fixed, and it is relatively easy for us to transfer knowledge and skills to coworkers. | 0.017 | 0.659 | -0.063 | -0.169 | 0.168 | -0.063 | 0.557 |  |  |
| Before the training, my boss expected that my awareness, knowledge, and skills would be improved by the training. | 0.107 | 0.644 | -0.002 | 0.087 | -0.112 | 0.076 | 0.468 |  |  |
| A third party usually checks whether people at my workplace practice what they learned in any training. | 0.054 | 0.641 | -0.215 | 0.022 | 0.345 | -0.214 | 0.363 |  |  |
| After the training, the working environment (e.g., tools and equipment) was in place for me to apply the knowledge and skills I had learned. | 0.196 | 0.624 | -0.187 | -0.058 | 0.118 | 0.248 | 0.409 |  |  |
| Before the training, my boss and/or colleagues reinforced my motivation for attending the training. | 0.056 | 0.613 | 0.170 | 0.007 | -0.090 | 0.031 | 0.507 |  |  |
| After the training, I had an opportunity to apply in my workplace what I had learned during the training. | 0.577 | 0.594 | -0.254 | -0.082 | -0.144 | 0.090 | 0.281 |  |  |
| During the training, my boss had a good impression of the contents of the training. | 0.058 | 0.593 | 0.036 | 0.098 | -0.122 | 0.176 | 0.493 |  |  |
| My workplace regularly evaluates the performance of each worker. | -0.190 | 0.586 | 0.174 | 0.088 | 0.074 | 0.145 | 0.514 |  |  |
| Community people (individuals or groups) usually support me in my work. | -0.079 | 0.564 | 0.003 | -0.063 | 0.318 | -0.138 | 0.536 |  |  |
| During the training, my boss and/or colleagues covered my tasks. | -0.299 | 0.521 | 0.402 | -0.084 | -0.015 | 0.066 | 0.614 |  |  |
| After the training, I had an opportunity in my workplace to look back on the implementation status (progress, achievements) of what I had learned during the training. | 0.582 | 0.518 | -0.224 | 0.075 | -0.116 | 0.094 | 0.246 |  |  |
| After the training, my colleagues cooperated with me when implementing what I had learned during the training. | 0.348 | 0.491 | -0.095 | 0.056 | 0.054 | 0.289 | 0.329 |  |  |
| After the training, I was able to spend enough time applying what I had learned during the training. | 0.443 | 0.462 | -0.109 | 0.068 | -0.003 | 0.120 | 0.396 |  |  |
| After the training, some people accepted and/or provided consultation for me when applying what I had learned during the training. | 0.234 | 0.430 | -0.105 | 0.079 | 0.171 | 0.391 | 0.363 |  |  |
| I could actively attend the training. | 0.001 | -0.002 | 0.798 | 0.023 | -0.209 | -0.012 | 0.381 | Encouraging learning environment | 0.92 |
| The training had an atmosphere where participants could talk freely, and any mistake was allowable. | 0.045 | -0.038 | 0.735 | 0.012 | 0.020 | -0.020 | 0.432 |  |  |
| I could attend the training in a relaxed mood. | 0.139 | 0.036 | 0.645 | -0.006 | -0.232 | 0.002 | 0.487 |  |  |
| The teaching skill level of lecturers (speaking speed, clear and well-designed explanations) was high enough. | 0.020 | -0.028 | 0.634 | 0.046 | -0.058 | 0.030 | 0.573 |  |  |
| During the training, lecturers gave feedback about my speech and behavior. | 0.244 | -0.144 | 0.601 | 0.003 | 0.002 | -0.190 | 0.495 |  |  |
| I exchanged information on the training content with other participants during the training. | 0.412 | 0.031 | 0.577 | -0.331 | -0.028 | -0.038 | 0.446 |  |  |
| The classroom environment was comfortable (temperature, humidity, cleanliness, noise and smell, etc.). | 0.070 | -0.063 | 0.567 | 0.127 | -0.052 | -0.053 | 0.592 |  |  |
| Lecturers had high expertise (knowledge, experience, and achievements) related to the training content. | -0.044 | 0.175 | 0.546 | 0.087 | -0.299 | -0.001 | 0.597 |  |  |
| Training materials (textbooks, handouts, videos, etc.) were easily understandable. | 0.246 | -0.123 | 0.545 | 0.057 | -0.087 | -0.089 | 0.550 |  |  |
| During the training, I participated in the training at the appropriate time for my career. | 0.075 | 0.177 | 0.534 | 0.070 | 0.049 | -0.200 | 0.500 |  |  |
| During the training, there was time to present my/our action plan. | 0.334 | 0.121 | 0.531 | -0.097 | -0.126 | -0.198 | 0.472 |  |  |
| During the training, there was more time for study tours than lectures. | 0.109 | 0.228 | 0.509 | -0.294 | 0.079 | 0.033 | 0.620 |  |  |
| At the end of the training, I could understand the training content well. | 0.409 | -0.065 | 0.508 | 0.006 | -0.175 | 0.065 | 0.405 |  |  |
| During the training, my boss and/or colleagues covered my tasks. | -0.147 | 0.054 | 0.487 | 0.182 | 0.052 | 0.381 | 0.485 |  |  |
| During the training, I made an effort to understand the training content. | 0.332 | -0.039 | 0.487 | 0.041 | -0.099 | 0.196 | 0.412 |  |  |
| The training period (days, hours) was appropriate. | 0.079 | -0.160 | 0.482 | 0.094 | 0.101 | 0.120 | 0.655 |  |  |
| During the training, the interpreter explained in plain words, and there was no problem with interpretation. | 0.033 | 0.063 | 0.457 | 0.054 | 0.025 | -0.168 | 0.714 |  |  |
| During the training, the training content was arranged to promote the step-by-step understanding of trainees. | 0.359 | -0.051 | 0.446 | 0.073 | 0.059 | 0.073 | 0.435 |  |  |
| The difficulty level of the training was appropriate for me during the training. | 0.250 | -0.050 | 0.424 | 0.097 | 0.013 | 0.326 | 0.425 |  |  |
| During the training, my boss and/or colleagues covered my tasks for me. | -0.299 | 0.521 | 0.402 | -0.084 | -0.015 | 0.066 | 0.614 |  |  |
| Before the training, I felt that the training would be helpful for my subsequent work. | 0.089 | -0.074 | -0.082 | 0.862 | -0.025 | -0.054 | 0.307 | Job-aligned training and readiness | 0.92 |
| Before the training, the needs of the workplace corresponded to the training contents. | 0.074 | -0.056 | -0.034 | 0.841 | 0.004 | 0.033 | 0.288 |  |  |
| Before the training, I had already perceived the need for the training that I would participate in. | 0.004 | 0.053 | 0.039 | 0.812 | -0.111 | -0.014 | 0.292 |  |  |
| Before the training, I had already understood what the benefit of the training was. | -0.028 | -0.002 | 0.053 | 0.791 | -0.007 | 0.185 | 0.285 |  |  |
| Before the training, I prepared myself for the training by reading materials and working on pre-assignments. | 0.055 | 0.092 | -0.113 | 0.751 | 0.168 | -0.164 | 0.338 |  |  |
| Before the training, I had already understood the objectives of the training. | -0.075 | -0.029 | 0.137 | 0.750 | 0.031 | 0.176 | 0.326 |  |  |
| Before the training, I received an explanation about the contents of the training in detail from my company or boss. | -0.118 | 0.050 | -0.072 | 0.663 | 0.081 | 0.166 | 0.560 |  |  |
| Before the training, I wanted to serve others' needs, such as health service beneficiaries (residents, patients) or relevant departments within my organization. | 0.289 | -0.001 | 0.038 | 0.590 | -0.069 | -0.168 | 0.418 |  |  |
| Before the training, I thought I was selected from many candidates, which increased my motivation for the training. | 0.055 | 0.027 | 0.106 | 0.571 | 0.086 | -0.442 | 0.410 |  |  |
| Before the training, I wanted to fulfill my role and be recognized by others. | 0.243 | -0.062 | 0.037 | 0.547 | 0.112 | -0.385 | 0.416 |  |  |
| Before the training, I wanted to gain knowledge and skills and enhance my capability (high motivation). | 0.333 | -0.079 | 0.095 | 0.538 | -0.212 | -0.189 | 0.441 |  |  |
| After the training, my boss expressed a negative attitude toward the application of what I had learned during the training. | 0.003 | -0.036 | -0.217 | -0.062 | 0.761 | 0.188 | 0.455 | Limited authority | 0.78 |
| After the training, I applied for a vacant position at an external organization or was headhunted by an external organization. | 0.045 | -0.092 | -0.171 | -0.066 | 0.737 | 0.065 | 0.509 |  |  |
| After the training, I did not have the authority to implement my/our action plan. | -0.165 | -0.086 | -0.099 | 0.174 | 0.698 | -0.042 | 0.523 |  |  |
| After the training, I exchanged information on the training content with other participants. | 0.179 | 0.259 | -0.053 | 0.095 | 0.194 | 0.569 | 0.356 | Altruistic desire | 0.54 |
| After the training, I exchanged information except the training content with other participants. | 0.238 | -0.065 | 0.048 | -0.085 | 0.418 | 0.440 | 0.562 |  |  |
| Before the training, I thought I was selected from many candidates, which increased my motivation for the training. | 0.055 | 0.027 | 0.106 | 0.571 | 0.086 | -0.442 | 0.410 |  |  |
| Before the training, I wanted to practice/implement what I would learn in the training and get promoted. | 0.121 | 0.159 | -0.043 | 0.382 | 0.292 | -0.462 | 0.428 |  |  |
